# Electroplating Composite Coatings of Nickel with Dispersed WO_3_ and MoO_3_ on Al Substrate to Increase Wear Resistance

**DOI:** 10.3390/ma18122781

**Published:** 2025-06-13

**Authors:** Petr Osipov, Roza Shayakhmetova, Danatbek Murzalinov, Azamat Sagyndykov, Ainur Kali, Anar Mukhametzhanova, Galymzhan Maldybayev, Konstantin Mit

**Affiliations:** 1 Republican State Enterprise National Center on Complex Processing of Mineral Raw Materials of the Republic of Kazakhstan, RSE NC CPMRM RK, Jandossov Str., 67, Almaty 050036, Kazakhstan; osipovapi@mail.ru (P.O.); twice777@mail.ru (A.S.); ainurkali99@mail.ru (A.K.); narike.91@gmail.com (A.M.); galimjan_87@mail.ru (G.M.); 2 Institute of Physics and Technology, Satbayev University, Almaty 050013, Kazakhstan; d.murzalinov@sci.kz (D.M.); k.mit@sci.kz (K.M.)

**Keywords:** nickel, tungsten oxides, molybdenum oxides, electroplated coatings, composite, dispersed phase, redox mechanism, paramagnetic particles

## Abstract

Investigations of the synthesis of multicomponent coatings and their subsequent application to metal substrates to increase the wear resistance of materials is relevant for industry. Nickel compounds obtained from oxidized magnesia-iron nickel ores with a desorption rate of more than 94% were used to create Ni-MoO_3_-WO_3_ electroplating. Such composite samples formed from an aqueous alcohol solution reduced the content of sodium and ammonium chlorides. The annealing and dehydration of samples at a temperature of 725 °C in an air atmosphere made it possible to achieve the highest level of crystallinity. In this case, an isomorphic substitution of W atoms by Mo occurs, which is confirmed by electron paramagnetic resonance (EPR) spectroscopy studies. The invariance of the shape of the EPR spectrum with a sequential increase in microwave radiation power revealed the stability of the bonds between the particles. The surface morphology of Ni-MoO_3_-WO_3_ films deposited on an Al substrate is smooth and has low roughness. In this case, an increased degree of wear resistance has been achieved. Thus, a recipe for the formation of an electroplating with stable bonds between the components and increased wear resistance was obtained.

## 1. Introduction

Determining the conditions for the formation of a dense and uniform packing of particles of more than two-component substances with a reduced defect level is promising for composite materials. Most natural objects have a compositional structure with hierarchical features. Due to the presence of several types of interactions at the atomic level, an important advantage of composite materials is their design flexibility. This makes it possible to synthesize new materials for specific applications with variations in components [1].

The development of research in the field of industrial materials has led to increased wear and corrosion resistance as well as resistance to elevated temperatures. In this regard, due to the above-mentioned enhanced characteristics, composite coatings with a metal matrix (MMCs) are becoming extremely important [2]. The properties of these materials are primarily determined by the phase composition of their components and the distribution and quantity of co-deposited grains [3,4,5].

The reinforcing elements of MMCs are mainly ceramic particles (oxides, carbides, nitrides, borides, etc.) containing ionic and/or covalent compounds, whereas metal matrices have a metallic bond between atoms [6]. The presence of several types of interactions between particles determines the wide variations in the properties of materials. As a result of the formation of covalent/ionic bonds, strength, Young’s modulus, and hardness are significantly increased. In addition, this reduces the deformability, electrical and thermal conductivity, and density of the substance [7,8,9,10].

Based on the dominance of the metal matrix in MMCs’ structures, the potential applications of such composites include all metal-related industries [6].

Low weight, high strength, and resistance to various impacts make Al and Ti widely used in the aerospace industry [11]. However, metals need to be strengthened to increase the level of safety during flight. Innovative MMCs can be used in the manufacture of such structural elements. For example, transformable wings that can change shape in flight contribute to increased aerodynamic efficiency and fuel economy. The development of active vibration control systems on this basis will lead to a reduction in vibration in aircraft and spacecraft. Moreover, MMC applications for satellite components as well as other specific applications are also promising [12,13,14]. Their use significantly increases the comfort of operation and reduces wear on parts [6].

Metal materials are the foundation of automotive constructions due to their strength, durability, etc. However, for more environmentally friendly production, the relevance of light and fuel-efficient vehicles is increasing. This has stimulated the application of high-strength light metals such as aluminum and magnesium. Further performance improvements are possible as a result of the use of composite materials. Such materials are especially important for the manufacture of engine components and braking systems, as well as other important parts of machine. Moreover, their application enhances crash resistance and the reliability of vehicles. Also, the reinforcement of composite materials with metal particles increases the corrosion resistance of car parts exposed to aggressive environments [15,16].

However, further development in the field of MMCs requires the adaptation of materials to important technological challenges. These include a lack of effective approaches to material design, limited composition control, and the need for cost-effective manufacturing methods [17,18,19,20].

One of the most effective methods for obtaining MMCs is galvanic deposition from heterogeneous electrolyte suspensions [21,22]. For this purpose, the use of compounds obtained directly after processing oxidized ores is promising. These compounds, purified from accompanying impurities and sequentially sorbed on special resins, are of high quality for the creation of electrolytes. Therefore, this method is relevant for a wide range of industrial applications.

Nickel with a high melting temperature and excellent corrosion resistance is extremely important for the manufacture of the hottest machine parts [23,24,25]. Despite this, the relatively low specific strength is the main disadvantage. At the same time, a composite based on a nickel matrix with the introduction of reinforcing phases increases the strength of the material [26,27,28].

Individual as well as mixed and co-deposited oxides of tungsten and molybdenum are widely used as phases of incorporation into the nickel matrix [29,30]. The presence of redox activity in molybdenum and tungsten trioxides stimulates their intensive growth [31,32,33,34]. This is due to the developed morphology of the surface of these particles and the presence of a high concentration of uncompensated charges. The non-compact structure of molybdenum trioxide crystals, which includes interlayer spaces, makes these compounds promising for the formation of antifriction properties of the galvanic surface. This significantly increases the wear resistance of metal-matrix composite materials [35,36].

Dispersed WO_3_ and MoO_3_ embedded in the nickel matrix during electrodeposition to form composite films lead to increased (up to 2–3 times) wear resistance compared with pure nickel [37]. The main advantage in this case, which determines their application, is the preservation of strength characteristics at elevated temperatures.

Ni-MoO_3_ composite coatings have greater wear resistance than Ni-WO_3_ ones. The stability of highly dispersed molybdenum trioxide in the electrolyte is much lower, and after electrodeposition, it develops surface relief [38,39]. Therefore, a mixture of tungsten trioxide and molybdenum is more suitable, but the properties obtained are not always additive.

Obtaining individual molybdenum and tungsten oxides is a technologically simple process. However, their co-precipitation in a given stoichiometric ratio is more complex. This is primarily due to the different formation time of the precipitate of molybdenum and tungstic acids. In addition, atoms of molybdenum and tungsten, due to their close size and electronegativity, can isomorphically replace each other. A solid solution of metal substitution is released during their precipitation [40]. In this case, a certain number of impurities in the form of sodium and ammonium compounds can also be trapped in the deposited substance.

Synthesis of a complex structure of a substance increases the probability of formation of structural defects. This is especially important for systems containing more than three components. Therefore, achieving the conditions for the synthesis of a structure with the isomorphic substitution of different particles does not lead to the formation of additional defects. In this case, a more densely packed structure of the substance is formed.

The formation of the interface between the metal matrix and the reinforcing components is important because it is a large part of the composite. This system is not in complete thermodynamic equilibrium. To transfer the load from the matrix to the reinforcement, the degree of bonding between them must be well-established. The interfacial bonding affects the mechanical, thermal, and other properties of MMCs. In this case, the defect formation and distribution of particles with an uncompensated charge plays a key role [41].

There are various methods for the synthesis of both individual and mixed oxides of W and Mo, mainly in the form of thin films: electrochemical deposition, thermal evaporation, and vapor deposition [42,43]; solvothermal synthesis [44]; gas deposition [45]; sol–gel [46]; carbonyl pyrolysis [47]; chemical and hydrothermal deposition [48,49,50]; etc.

The following three groups of factors influence the electrodeposition process and, consequently, the microstructure of the composite coating: electrolysis conditions (presence of additives, composition of the electrolytic bath, pH and temperature changes, electrolyte mixing), current conditions (current type and density), and characteristics of reinforcing grains (size, concentration, surface characteristics) [2].

- Statement of problem:

An urgent direction in the development of research on multicomponent composite materials, especially for the synthesis of coatings based on them, is to increase wear resistance. At the same time, the large number of components causes a high level of defects in the substance. Obtaining structures under conditions of isomorphic substitution of atoms of various components makes it possible to reduce the level of defects and consequently increase the wear resistance of the substance.

- Goal:

Investigation of the synthesis of the Ni-MoO_3_-WO_3_ composite structure with a dense packing of particles and isomorphic substitution of Mo atoms by W to increase the wear resistance of the material.

- Novelty:

The use of 20% H_2_SO_4_ in a technologically simplified metallurgical method made it possible to obtain high-purity nickel for the metal matrix of the composite.

For the first time, the transition of Ni-MoO_3_-WO_3_ matter from an amorphous state to a crystalline one, including the isomorphic substitution of Mo atoms for W atoms, was carried out as a result of annealing structures in an air atmosphere at 725 °C. This transformation does not lead to the formation of excessive structural defects and increases the wear resistance of the material.

## 2. Materials and Methods

### 2.1. Materials and Synthesis

#### 2.1.1. Metallurgical Process of Nickel Production

**Figure 1:**
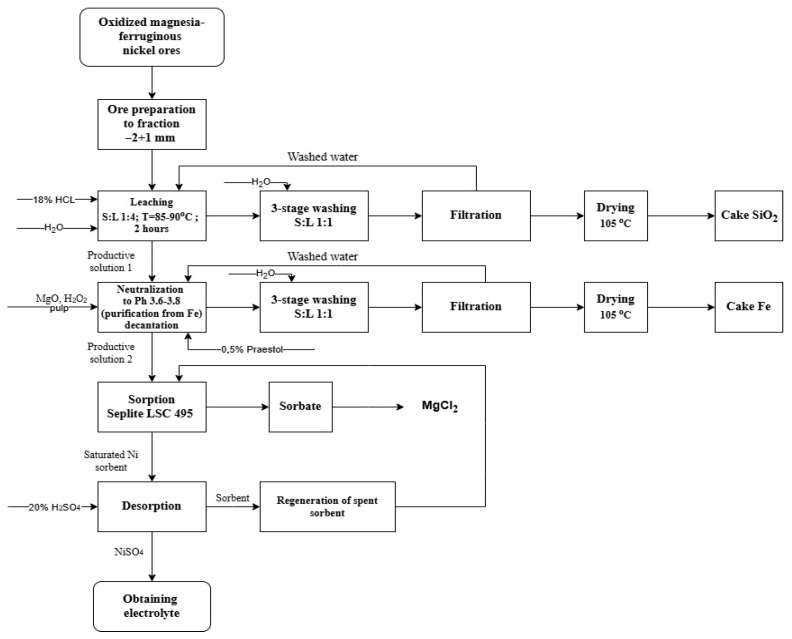
Scheme of the metallurgical process of nickel production.

The metal matrix plays a key role in the structure of MMCs. Therefore, in this article, the metallurgical step-by-step process of obtaining nickel compounds with increased purity is considered separately. A promising sorption technology was used to extract nickel from oxidized magnesia—ferruginous nickel ores [51,52]. This process includes the stages of raw material preparation, acid leaching, solution purification, and the sorption and desorption of nickel (Figure 1). At the first stage, the ore is crushed to a fraction of −2 + 1 mm, then leached with 18% HCl at a temperature of 85–90 °C (solid–liquid—1:4) within 2 h. After three-stage washing and filtration, a silica residue (SiO_2_ cake) is released. Further, the productive solution is purified from iron by precipitation at pH 3.6–3.8, followed by a three-stage washing and drying of the precipitate (Fe cake). Nickel sorption was carried out from a purified solution using Seplite LSC 495 ion exchange resin. After that, the sorbent saturated with nickel undergoes desorption by passing through a 20% H_2_SO_4_ solution. This ensures that a high level of nickel is released into the solution. The spent sorbent undergoes regeneration and can be reused, which increases the economic efficiency of the process.

The use of this technology can significantly reduce the cost of manufactured products. This is due to the almost complete conformity of desorbate as the base for the electrolyte. Most of the electrolytes used in practice are obtained by dissolving nickel sulfate powder in water. Obtaining nickel sulfate powder from a similar desorbate requires additional technological operations: desorbate evaporation, crystallization of nickel sulfate, separation of the target product, and drying. Moreover, the dehydration of the substance, in this case, occurs with high energy consumption [53]. The method proposed in this article does not provide for such a large number of process stages and energy consumption. Therefore, the application of this method makes it possible to achieve technological efficiency.

The following reagents were used for metallurgical nickel production processes. Oxidized magnesia—ferruginous nickel ores from the “Gornostayevskoye” deposit—were used as initial raw materials. Hydrochloric acid (37% HCl), supplied by JSC “Kaustik Company” (Pavlodar, Kazakhstan), was used as the leaching agent in the experiments. Sigma-Aldrich (St. Louis, MO, USA) is a manufacturer of hydrogen peroxide (H_2_O_2_, 37%) and magnesium oxide (MgO). The flocculant Praestol 2500 (Ashland Inc., Wilmington, DE, USA) was used without any pre-treatment. The ion exchange resin LSC495 (bispiolamine, Lanxess Deutschland GmbH, Cologne, Germany) was used in the sorption and desorption processes. Sulfuric acid (96% H_2_SO_4_, JSC “Base No. 1 of Chemical Reagents”, Moscow, Russia) was used in the desorption process. All experiments were conducted using distilled water.

#### 2.1.2. Synthesis of Ni-Mo-W Electroplating and Subsequent Deposition on the Al Substrate

It is known [54,55] that aqueous alcohol solutions dissolve monovalent metals well, and the solubility of other metals in them is significantly lower. Therefore, in order to reduce the content of NaCl and ammonium chloride, W-Mo compounds were co-precipitated with hydrochloric acid from aqueous alcohol solutions. Further, the samples were washed with aqueous alcohol solutions on a filter.

Nickel sulfate solutions were prepared by adding the required components in stoichiometric proportions to achieve the following electrolyte composition: NiSO_4_—300 g/L, NiCl_2_—60 g/L, and H_3_BO_3_—30 g/L (pH = 4.04).

For the dispersed phase, either individual molybdenum trioxide or tungsten–molybdenum oxide components, obtained by co-precipitation from aqueous alcoholic solutions, were used. The WO_3_/MoO_3_ ratio was 0.2 at a pH of 0.5–1.0. The WO_3_/MoO_3_ ratio was 0.3 at a pH of 1–1.5. The WO_3_/MoO_3_ ratio was 0.5 at a pH of 2–3. The concentration of the dispersed oxide phase suspended in the deposition electrolyte was maintained at 20 g/L.

Sodium tungstate hexahydrate and ammonium molybdate tetrahydrate (both components are “chemically pure”) were used as initial materials for the dispersed phase. These compounds were dissolved in distilled water and precipitated with hydrochloric acid. After that, all samples were separated from the mother liquor via filtration and washed on the filter using either water or an aqueous alcoholic solution. Then, the samples were gradually heat treated at 120 °C, 420 °C, and 600 °C for 1 h in an air atmosphere. The final heat treatment was carried out to crystallize the structures at temperatures of 700 °C and 725 °C for 1 h in an air atmosphere.

The electrolytic deposition of Ni-MoO_3_-WO_3_ via electroplating onto an aluminum substrate was carried out at a cathode current density of 2 A/dm^2^.

### 2.2. Characterization Methods

DTG, DTA, and TG analyses were performed using a Derivatograph Q-1500D (Paulik-Erdey, Budapest, Hungary).

The dimensional and morphological characteristics of the dispersed phase particles, as well as the microrelief of the composite surfaces, were analyzed using a scanning electron microscope JSM-6490LA (JEOL, Akishima, Japan). The analytical working distance was 10 mm, the take-off angle was 35°, and the high resolution was 3.0 nm. An analytical scanning electron microscope JSM 6490 LA (JEOL, Akishima, Japan), outfitted with the JED 2300 EDS spectrometer, was used to perform the elemental analysis of the materials.

The structure of the samples was studied by X-ray diffraction (XRD) on a Rigaku MiniFlex 600 X-ray spectrometer with copper radiation (CuK-α) with the following conditions: X-ray tube voltage 40 kV, tube current 15 mA, goniometer movement step 2θ = 0.02°.

A JES-FA 200 (JEOL, Akishima, Japan) EPR spectrometer was used to confirm the hypothesis of the isomorphic substitution of paramagnetic particles as a result of the annealing of structures. Measurements were taken at ~9.4 GHz (X-Band) and ~35 GHz (Q-Band), with a microwave frequency stability of ~10^−6^ and sensitivity = 7 × 10^9^/10^−4^ Tl. The resolution was 2.35 μT. The output power ranged from 200 mW to 0.1 μW, with a Q-factor of 18,000. The signal was detected in the microwave radiation power range from 0.5 to 2 mW. To achieve high accuracy in the analysis, the following parameters for obtaining spectra were selected: time constant—0.1 s, signal modulation amplitude—0.4 mT, time of one scan—2 min, number of spectrum accumulations—4, signal gain factor—1400.

The wear resistance of the composites was evaluated using an SMTs-2 friction machine (Russia, Rybinsk city, Rybinsk State Aviation Technical University) under dry friction conditions without lubrication. This parameter was quantified as the ratio of wear scar depth to the friction path length (a dimensionless parameter). A load of 1.1 kg/cm^2^ was applied to the samples for 2 h. Before the wear test, the samples were run until a constant moment of friction was reached. The wear was assessed by the gravimetric method. The error of the device for measuring wear resistance is less than 5%.

## 3. Results

### 3.1. Obtaining Nickel as a Matrix Metal in a Composite

The main basis of an MMC is a metal matrix, the purity of which determines the properties of the composite. The method proposed in this article for obtaining nickel as a matrix material eliminates numerous technological steps. The use of 20% sulfuric acid in this case ensured a high level of desorption of nickel from magnesia–ferruginous nickel ores of 94%. The results of the nickel production processes are presented in Table 1.

The obtained nickel had a high degree of purity and met the requirements necessary for use in electrodeposition processes.

### 3.2. Investigation of the Process for Obtaining Phases of Tungsten and Molybdenum Oxides Embedded in a Nickel Matrix

The next step in the formation of an MMC is to obtain dispersed phases of MoO_3_ and WO_3_ in the structure of the Ni matrix. In this case, a dense packing of particles should be formed, ensuring the strength of the material.

**Figure 2:**
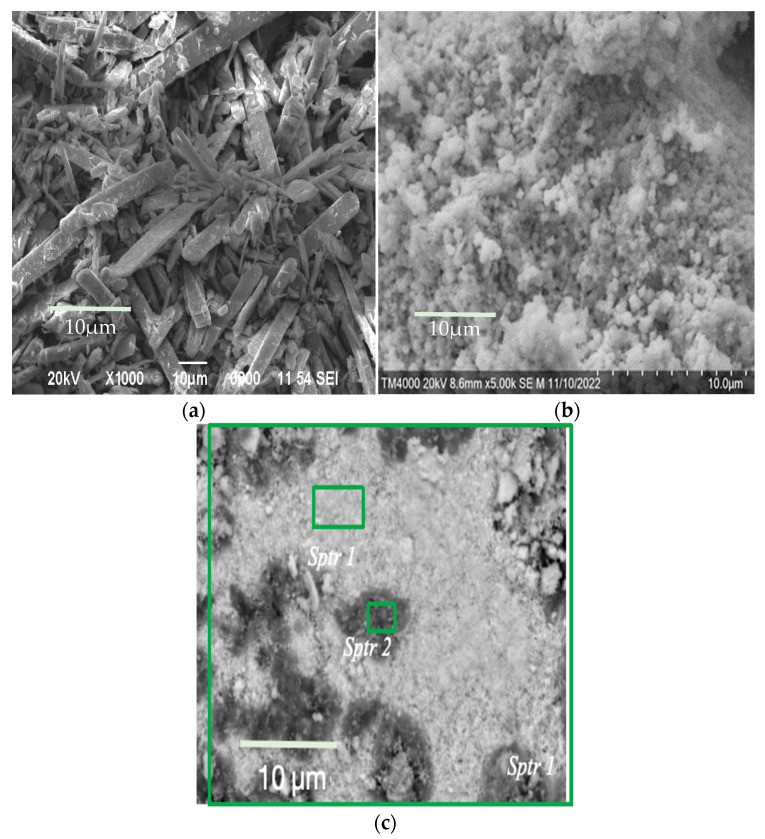
SEM images of the morphology of (**a**) molybdenum, (**b**) tungsten, and (**c**) co-precipitated tungsten–molybdenum compounds obtained from aqueous alcohol solutions with a W/Mo molar ratio of 0.2.

SEM images of the surface of the obtained samples are shown in Figure 2.

Molybdenum compounds precipitated have the form of large, elongated, flat crystals (Figure 2a). At the same time, individual compounds of tungstic acids have a coarse-grained form (Figure 2b).

Co-precipitated tungsten–molybdenum compounds have a dense packing of particles. The absence of small particles of tungsten compounds is noticeable (Figure 2c). This indicates the same rate of synthesis for tungsten and molybdenum compounds under these conditions. The figure shows dark spots on a light background, which is associated with the formation of lighter elements in high concentrations. The overall composition, as well as the compositions of the dark and light fragments of this sample (both untreated and washed after dehydration), are listed in Table 2. Since oxygen is a light element, its quantitative identification by the EDS method is difficult. Therefore, the presence of oxygen in the substance was determined in these studies.

**Figure 3:**
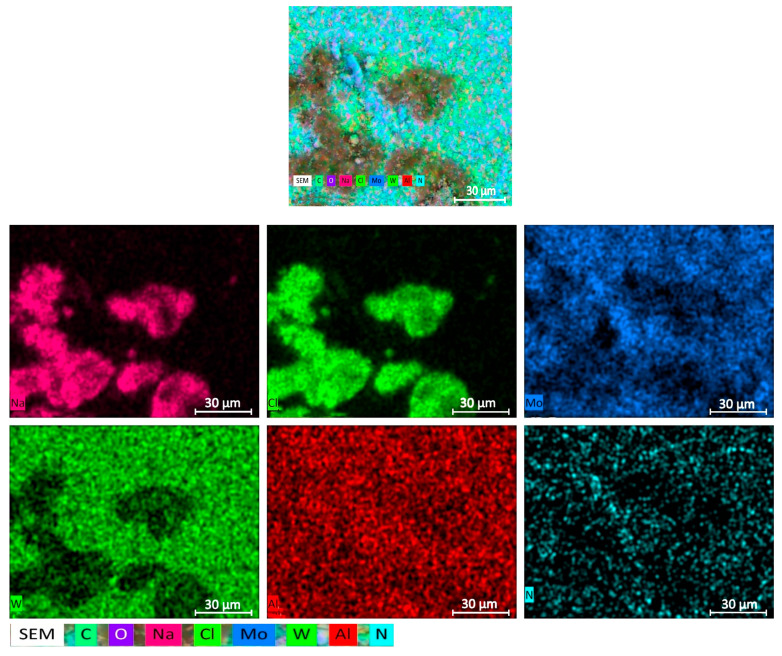
Elemental mapping of the sample with a W/Mo ratio of 0.2 obtained via aqueous alcohol precipitation.

Table 2 clearly shows the increased concentration of chlorine and sodium in the dark spots, while the light regions exhibit higher concentrations of tungsten–molybdenum compounds. A more detailed elemental composition of a sample with a W/Mo ratio of 0.2 obtained from aqueous alcohol solutions is shown in Figure 3.

The tungsten compounds in samples obtained from aqueous alcohol mixtures exhibit a well-defined and compact distribution. The distributions of molybdenum and tungsten correlate well.

The arrangement of chlorine and sodium atoms on the map almost coincides, so presumably these elements are in the form of sodium chloride. Their compact distribution, which repeats that of tungsten, indicates the encapsulation of NaCl inside the growing compounds of W. Therefore, the sodium chloride was not removed by the washing liquid. Additional washing with aqueous alcohol solutions of these crushed samples after drying and dehydration leads to a significant decrease in the concentration of sodium chloride (Table 2).

It should be noted that there is an absolute difference in the distributions of nitrogen and chlorine. At the same time, the distribution of nitrogen and the fragmentary distribution of molybdenum are basically the same. It follows that, with this precipitation method, the formed ammonium chloride is almost completely washed out of the sample.

### 3.3. Studies of the Processes of Heat Treatment and Dehydration of MoO_3_-WO_3_ Structures

**Figure 4:**
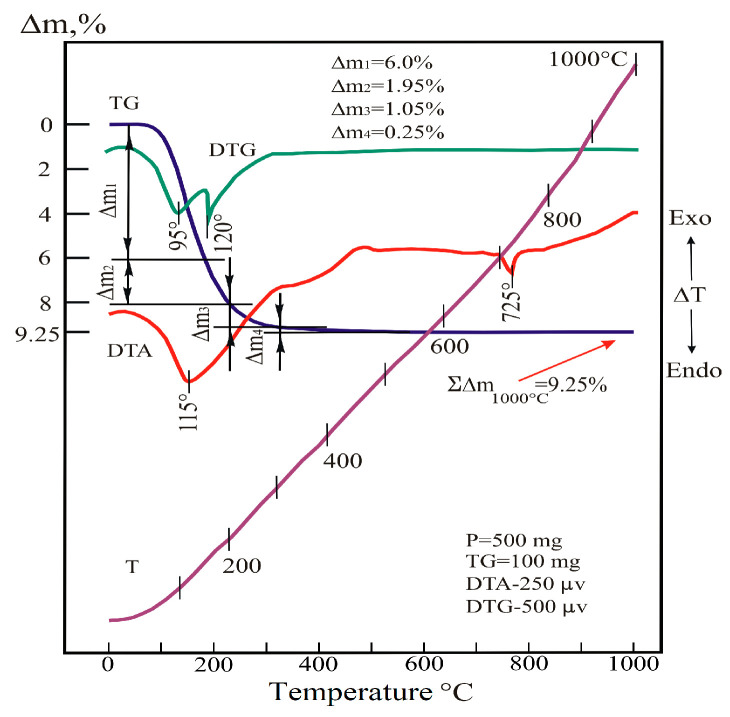
Derivatogram of a sample of co-deposited fine powders of tungsten and molybdenum oxides (red line—DTA, green line—DTG, blue line—TGA, purple—function of temperature).

The formation of a stable structure with certain properties occurs during the heat treatment of the substance. Therefore, the conditions of this process are very important. The process of the dehydration of the substance was studied using DTA, DTG, and TGA analyses. The air-dried powder WO_3_-MoO_3_ sample with a W/Mo ratio of 0.2 was examined under a continuous temperature rise from 20 to 1000 °C. The results of these studies are shown in Figure 4 and Table 3.

Thermal reactions occurring up to 500 °C were caused by the release of water molecules and hydroxyls partially bound to acid residues from the system. In the range of 20–200 °C, the main stage of dehydration occurred, which proceeds in two stages. The first stage (step, part) of dehydration (DTG peak is in the range of 95 °C) is carried out in the range of 20–140 °C. At the same time, the weight loss of the sample reaches 6%. The second stage (step) (DTG peak—in the vicinity of 120 °C) occurs in the range of 140–200 °C, where the mass of the sample decreases by another 1.95%.

The step-by-step mechanism of the release of H_2_O molecules from the system is determined by the characteristics of the bonds between the water molecules and the sample particles. The bond of the first portion of water particles with acid residues was low. The similar dependence for the second portion of water is higher, which led to an increase in the duration of the dehydration process.

At temperatures above 200 °C, the process is carried out by releasing the hydroxyl part of the water. In the range of 200–305 °C, intensive dehydration leads to a 1.05% weight loss in the sample. In the range of 305–545 °C, a 0.25% mass loss occurs in the sample.

The peak at 725 °C on the DTA curve is due to a low-intensity endothermic effect in the dehydrated system. Since this occurs without changing the weight of the sample, it should be attributed to the polymorphic transformation in the heated substance.

**Figure 5:**
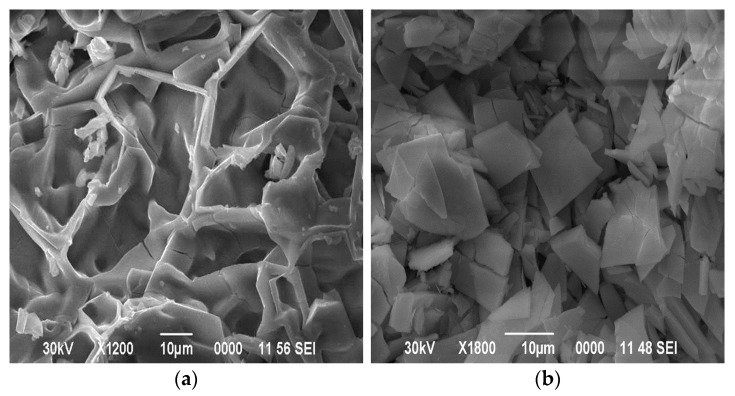
SEM images of the surface morphology of samples of co-deposited oxides of tungsten and molybdenum, heat-treated at (**a**) 600 °C and (**b**) 725 °C.

These studies showed noticeable differences in the structure of the samples obtained before and after polymorphic transformation (Figure 5).

A surface with an uneven particle distribution is noticeable after annealing the sample at a temperature of 600 °C. On the contrary, heat treatment at 725 °C resulted in the formation of a surface with a more uniform and compacted particle distribution. Thus, in order to obtain samples of co-deposited tungsten and molybdenum oxides, it is necessary to carry out dehydration and heat treatment at 700–725 °C.

Further, the precipitated powders obtained after dispersion and heat treatment were investigated by EPR and XRD analysis. After that, composite electroplating coatings based on a Ni metal matrix with a dispersed phase of MoO_3_ and WO_3_ were obtained by electrolysis.

### 3.4. XRD Studies

**Figure 6:**
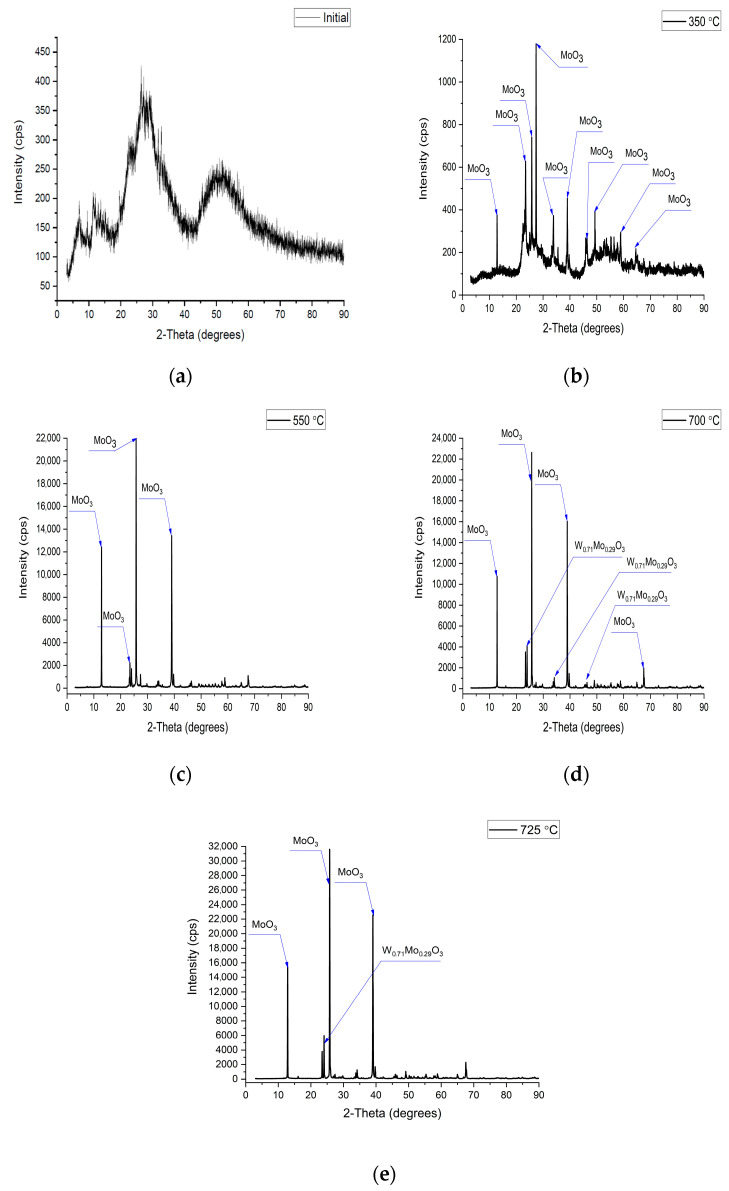
XRD spectra of (**a**) the initial sample, and samples after annealing at (**b**) 350 °C, (**c**) 550 °C, (**d**) 700 °C, (**e**) 725 °C.

Synthesis and subsequent heat treatment led to significant changes in the structure of the multicomponent substance. These transformations were determined by the XRD method (Figure 6). The growth of the intensity and a narrowing of the peak with increasing annealing temperature are noticeable from the spectra. This indicates an increase in the level of the crystallinity of the substance in this process.

When the annealing temperature increased to 725 °C, Mo and W atoms were substituted (Table 4). This process initiates when the temperature rises from 350 °C to 550 °C and reaches 71% at 700–725 °C.

An increase in the intensity of the peak of the tungsten–molybdenum oxides phase is noticeable with an increase in the annealing temperature (Figure 6d,e). The crystallinity level in this case also increased from 77% (550 °C) to 96% (725 °C), and the parameters of the crystal lattice became approximately the same (Table 5). This indicates the formation of a stable structure of this phase after annealing at 700–725 °C.

An increase in the crystallinity of a substance affects an increase in the wear resistance of the substance. This leads to an extension in the service life of devices based on this material. In the next section, the features of the substitution of Mo and W atoms are clarified.

### 3.5. EPR Studies

Determining the substitution of different atoms in a substance’s structure is a challenging process. For this purpose, a method with increased accuracy when measuring the parameters of paramagnetic particles should be applied. It was assumed that, in the case of non-isomorphic substitution of particles, additional defects with paramagnetic properties are formed. The EPR spectroscopy spectrum characterizes the distribution of such particles in the sample volume.

The following spectrum recording parameters made it possible to detect various signals with high accuracy. Reducing the amplitude of the modulation of the signal (0.4 mT) and the time constant (0.1 s) allows the system to manifest signals with low intensity. Accumulating the spectrum four times resulted in noise reduction by averaging.

**Figure 7:**
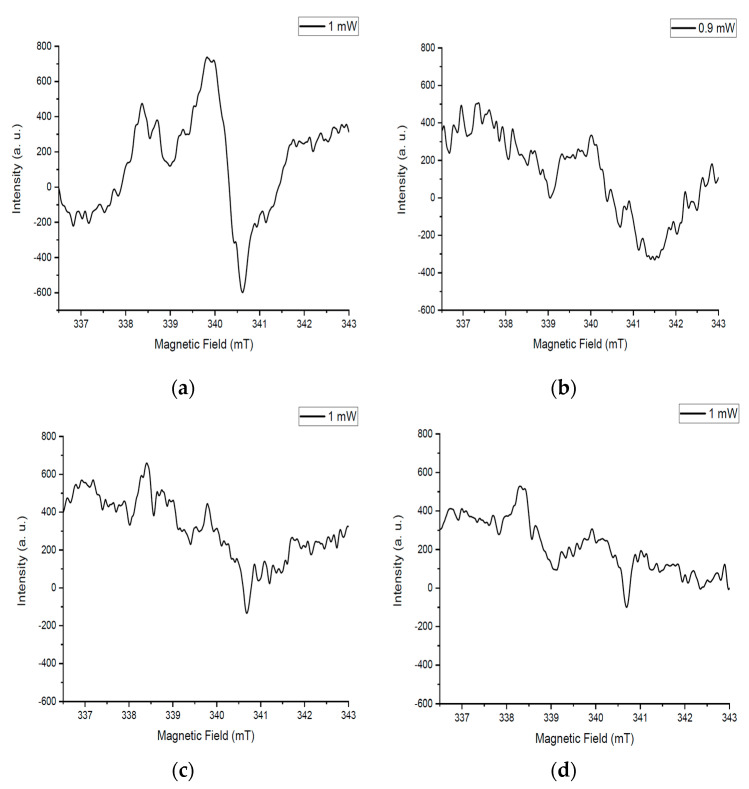
EPR spectra of samples after heat treatment: (**a**) 350 °C, (**b**) 550 °C, (**c**) 700 °C, (**d**) 725 °C.

An asymmetric intense signal is noticeable for the sample after annealing at 350 °C (Figure 7a). The amorphous structure of this sample contains a large concentration of particles with an uncompensated charge. Based on this, the intensity of the spectrum is high. The structure of the amorphous substance is heterogeneous, so the spectrum is not symmetrical.

The intensity of the signals for the sample after annealing at 550 °C decreased compared to the initial spectrum (Figure 7b). This is due to the ordering of the structure of the substance as a result of annealing at a higher temperature. The concentration of amorphous matter in the sample volume is relatively high, so the spectrum is not symmetrical.

The spectrum on Figure 7c has an even lower intensity, which is due to the crystallization of the substance as a result of annealing at 700 °C. The relative symmetry of the spectrum is noticeable, which is associated with a more uniform distribution of matter.

The temperature treatment at 725 °C is of key importance for the entire process. The signal in Figure 7d is the least intense, which is due to the highest ordering of the structure and, consequently, the low concentration of particles with an uncompensated charge. The symmetry of the spectrum is related to the following: when particles are non-isomorphically substituted, the concentration of defects increases, which manifest themselves in the symmetry of the EPR spectrum. In our case, this does not happen, which confirms the isomorphic substitution of tungsten particles for molybdenum.

**Figure 8:**
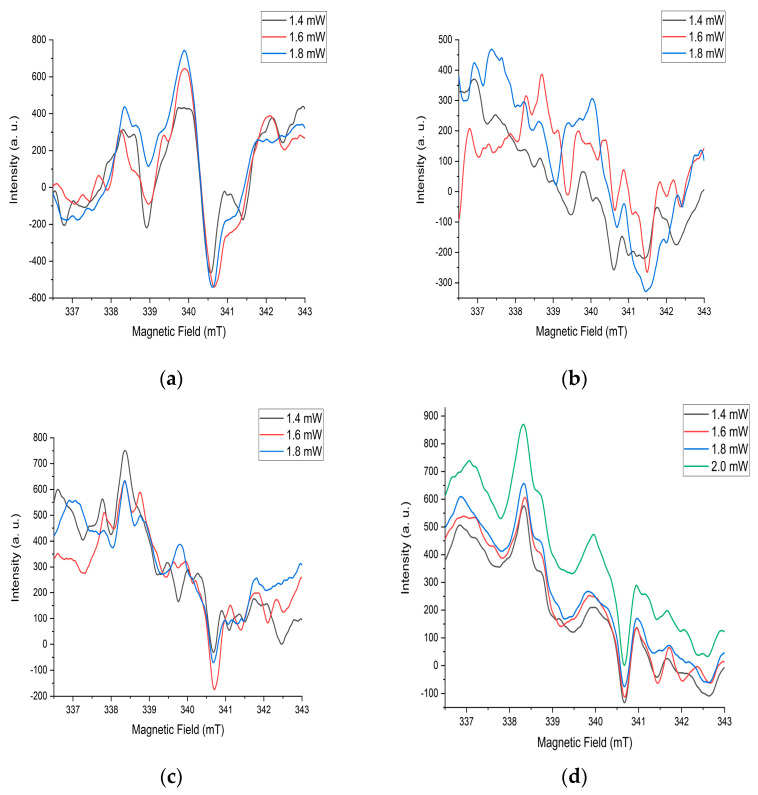
EPR spectra of the sample after annealing at (**a**) 350 °C, (**b**) 550 °C, (**c**) 700 °C, (**d**) 725 °C.

EPR signals from various paramagnetic particles manifest at different values of microwave radiation power. These values for Ni-MoO_3_-WO_3_ samples were from 0.2 to 2 mW. The constancy of the waveform with a sequential change in microwave radiation power during measurements indicates the stability of the structures after annealing at 725 °C (Figure 8d). Changes in the shape of the spectrum with an increase in power for other heat treatment modes also confirms this assumption (Figure 8a–c). This is closely related to the high level of crystallinity and energy stability of the substance after annealing at a temperature of 725 °C. There are several different interconnected signals in the spectrum. The diversity of the nature of signals is based on the difference in their shape in the spectrum. In this case, the relationship of the signals is determined by the same changes with successive increases in microwave power (Figure 8d).

### 3.6. Investigation of the Formation of Ni-MoO_3_-WO_3_ Galvanic Coatings on an Aluminum Substrate

**Figure 9:**
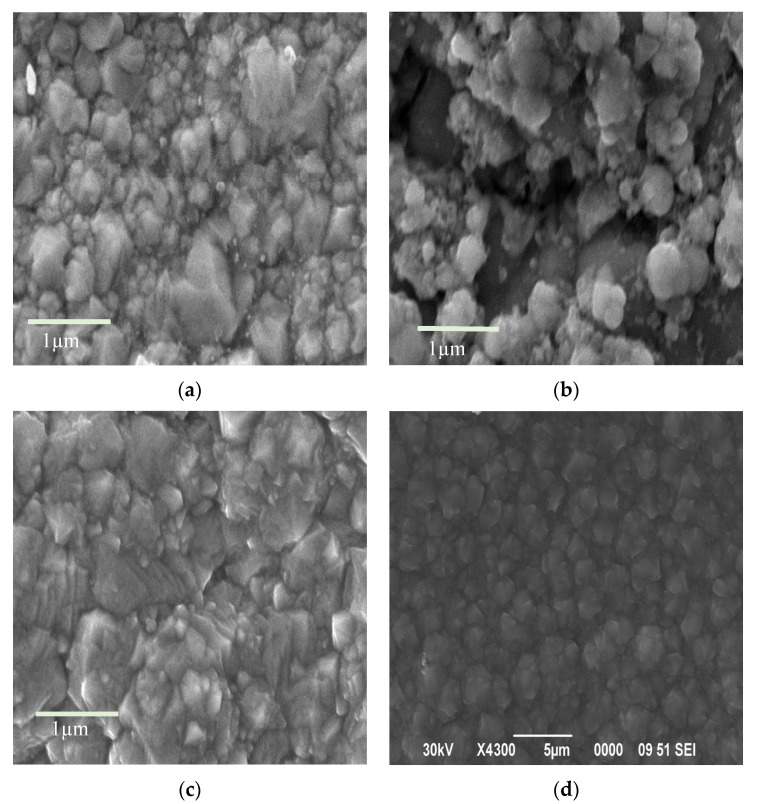
SEM images of the surface of electroplated coatings obtained using nickel electrolytes: (**a**) without a dispersed phase, resolution 1 µm; (**b**) containing molybdenum oxide as the dispersed phase, resolution 1 µm; (**c**) containing co-precipitated molybdenum–tungsten oxides, resolution 1 µm; (**d**) containing co-precipitated molybdenum–tungsten oxides, resolution 5 µm.

**Figure 10:**
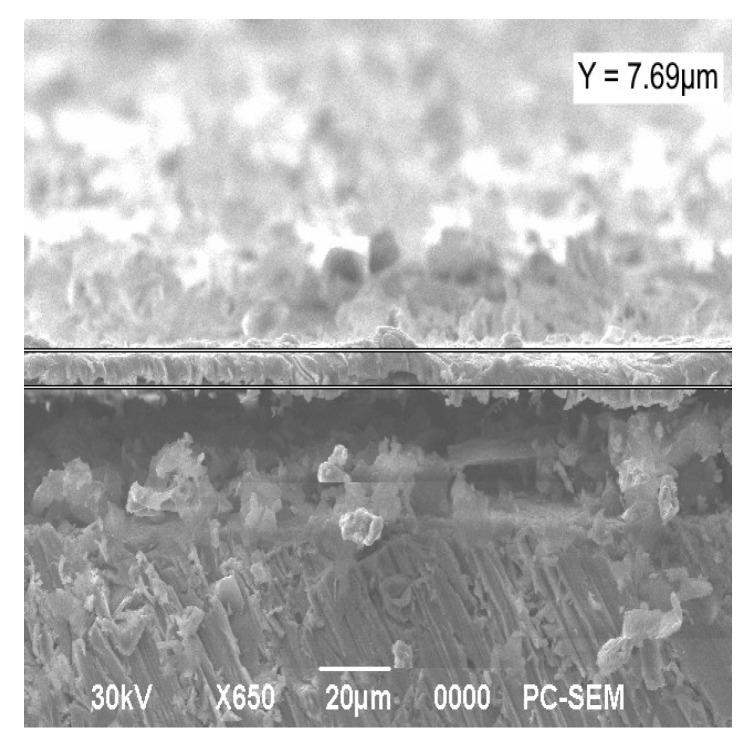
The cross-section of the Ni-MoO_3_-WO_3_ coating deposited on an Al substrate (resolution 20 µm).

The formation of composite coatings on a metal substrate is aimed at improving the quality characteristics of the substance. Structures with high stability obtained as a result of synthesis and subsequent heat treatment were used to form electroplating coatings by electrolysis. In this case, nickel electrolytes with a dispersed phase of molybdenum oxide or co-precipitated oxides of molybdenum and tungsten were applied. The surface morphology of the deposited Ni-MoO_3_-WO_3_ coatings is shown in Figure 9. The average coating thickness (h) was calculated from ten SEM cross-sectional images of the sample, h = 6.61 ± 0.51 µm (Figure 10).

The surface morphology of composite films obtained by the co-deposition of nickel and MoO_3_/WO_3_ particles has a similar structure to electroplated nickel. These films are characterized by a relatively smooth surface with roughness sizes below one micrometer. The electrolytic co-deposition of nickel with dispersed molybdenum trioxide results in a highly developed surface structure with significant roughness (Figure 9b). This increased roughness is attributed to the high redox activity of molybdenum trioxide.

The partial isomorphic substitution of molybdenum trioxide by tungsten trioxide reduces the redox activity of the resulting compound. This leads to more favorable conditions for deposition, resulting in a more homogeneous and smoother microrelief in the composite galvanic coatings (Figure 9c,d).

### 3.7. Wear Resistance Studies

**Figure 11:**
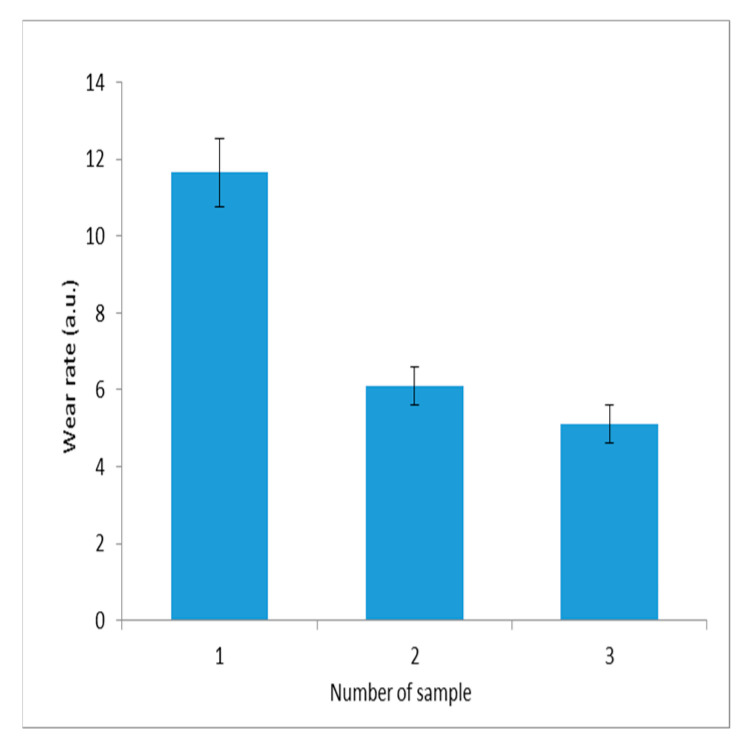
Wear rate of composite electrolytic coatings under boundary friction with a specific load of 7.5 MPa: 1—nickel; 2—Ni-MoO_3_ from aqueous alcohol solutions; 3—Ni-WO_3_/MoO_3_ from aqueous alcohol solutions.

The wear resistance of composite electroplating coatings is a critical factor in determining the durability of operation. It was evaluated using tribometer technology. The wear intensity of electrolytic composite coatings containing dispersed tungsten and molybdenum oxides embedded within the nickel matrix under boundary friction conditions is presented in Figure 11.

The wear values for the three samples are 11.65 ± 0.9 a.u., 6.1 ± 0.5 a.u., and 5.1 ± 0.5 a.u., respectively.

Figure 11 demonstrates that coatings containing tungsten–molybdenum compounds as the dispersed phase exhibit the highest wear resistance. These, as well as the antifriction properties of the composite material, are due to the layered structure of molybdenum oxide.

In contrast, the reduced wear resistance observed in samples containing individual molybdenum trioxide as the dispersed phase is attributed to the formation of a highly developed surface with increased roughness. This is the result of the increased redox activity of molybdenum trioxide.

## 4. Discussion

This section discusses the causes of the formation of a surface with a dense packing of coating particles during the isomorphic substitution of W and Mo. In aqueous alcohol solutions, especially at high pH, the formation of hydrocomplexes (WO_2_OH^+^, WO_2_(OH)_2_) is difficult. Hydrocomplexes can capture Na and Cl particles, and therefore the rate of formation of tungsten components decreases. In our work, almost all tungsten compounds are monomeric tungstate ions. This led to the substitution of W and Mo particles and the almost complete absence of a finely dispersed tungsten phase. In this case, a compact structure of the surface of the substance is formed.

The signs of isomorphic substitution of Mo and W particles during the transformation of the sample structure were determined by several methods. Elemental mapping revealed a coincidence of particle distributions. The DTA method determined the transformation of the structure without weight change during the dehydration of the substance. These results also indicate a compaction of the structure of the substance.

The consistent application of the following methods made it possible to confirm the transformation of the sample structure after annealing at 725 °C with the isomorphic substitution of Mo and W particles. The XRD method proved the substitution of particles, and EPR spectroscopy revealed the isomorphic nature of these structural changes.

## 5. Conclusions

In this work, a method for forming a WO_3_-MoO_3_-Ni coating on an Al substrate with increased wear resistance was developed. The use of a special metallurgical method for extracting nickel from ore made it possible to obtain a metal with increased purity for the formation of a composite matrix. Also, the application of this method ensured the technological efficiency of the process by reducing the number of stages. The synthesis of Ni-MoO_3_-WO_3_ structures from an aqueous alcohol solution made it possible to reduce the content of sodium and ammonium chlorides in the samples. At the same time, the rate of formation of the Mo and W components is approximately the same due to the absence of capture of Na and Cl particles by the tungsten components. Heat treatment at 725 °C in an air atmosphere led to an increase in the crystallinity of the samples. In this case, a dense packing of surface particles was formed by, in particular, the isomorphic substitution of W atoms for Mo. The Ni-MoO_3_-WO_3_ composite coating deposited on an aluminum substrate has low roughness and high wear resistance. The use of XRD and EPR methods made it possible to determine the isomorphic substitution of Mo and W particles, which led to the compaction of surface particles. Thus, the application of the structures studied in this work makes it possible to manufacture elements of mechanisms with high resistance to loads. This will ensure the development of many industries.

## Figures and Tables

**Table 1:** Solution composition.

Product	Ni Content, g/L	Fe Content, g/L	Mg Content, g/L
Solution after leaching	2.10	25.1	35.3
Solution after impurity removal for sorption	2.15	-	40.4
Desorbate	65.0	-	-

**Table 2:** Composition of various fragments of tungsten–molybdenum compounds obtained via co-precipitation from aqueous alcohol solutions, wt%.

Element	N	Na	Cl	Mo	W
Spectrum total	4.41	1.15	1.39	43.91	14.54
Spectrum 1	3.86	8.69	9.61	37.68	11.22
Spectrum 2	4.54	0.09	0.14	44.07	14.44
Sample after washing	4.35	0.04	0.07	45.15	13.82

**Table 3:** Thermogravimetric characteristics of the sample annealed in the range of 20–1000 °C.

Stages of the Process	Weight Loss Sequence	Weight Loss, %	Volatile Components	Temperature Ranges, °C
1	Δm1	6.0	H_2_O	20–140
2	Δm2	1.95	OH+ acid residue	140–200
3	Δm3	1.05	OH	200–305
4	Δm4	0.25	CO_2_	305–545
Total	∑Δm	9.25	H_2_O, OH, CO_2_	20–1000

**Table 4:** Crystal phases of the samples.

Temperature °C	The Crystalline Phase		
Initial	MoO_3_	WO_3_	-
350	MoO_3_	WO_3_	-
550	MoO_3_	WO_3_	W_0.4_Mo_0.6_O_3_
700	MoO_3_	WO_3_	W_0.71_Mo_0.29_O_3_
725	MoO_3_	WO_3_	W_0.71_Mo_0.29_O_3_

**Table 5:** Lattice parameters for the tungsten–molybdenum oxide phase.

Temperature °C	a (Å)	b (Å)	c (Å)
550	5.318335	5.524896	7.141768
700	7.442842	7.420851	7.609771
725	7.443129	7.421137	7.610064

## Data Availability

The original contributions presented in this study are included in the article. Further inquiries can be directed to the corresponding author.
